# Patient perspective on the management of atrial fibrillation in five European countries

**DOI:** 10.1186/1471-2261-13-108

**Published:** 2013-12-01

**Authors:** Ameet Bakhai, Anna Sandberg, Thomas Mittendorf, Wolfgang Greiner, André MS Oberdiek, Patrizia Berto, Edith Franczok, Trudie Lobban, Jose L Zamorano

**Affiliations:** 1 Barnet and Chase Farm Hospitals NHS Trust, The Ridgeway, Enfield, EN2 8JL Middlesex, UK; 2 AMORE Health Ltd, 11 Pinewood Avenue, HA5 4BN Pinner, UK; 3 Daiichi Sankyo Europe GmbH, Zielstattstrasse 48, 81379 Munich, Germany; 4 Herescon GmbH, Health Economic Research and Consulting, Herescon GmbH, Königsworther Str. 2 D, 30167 Hannover, Germany; 5 Health Economics and Health Care Management, University of Bielefeld, Bielefeld, Universitätsstraße 25, 33615 Bielefeld, Germany; 6 University of Padova, Padova, Via 8 Febbraio, 2-35122 Padova, Italy; 7 Analytica-Laser, Vicolo Stella 6, 37121 Verona, Italy; 8 Harris Interactive AG, Beim Strohhause 31, 20097 Hamburg, Germany; 9 Atrial Fibrillation Association, Chew Hill, Chew Magna, Avon BS40 8WB Bristol, UK; 10 Hospital Ramón y Cajal, University Alcala de Henares, Carretera de Colmenar Km 9.100, 28034 Madrid, Spain

**Keywords:** Atrial fibrillation, Arrhythmia, Patient satisfaction, Stroke, Anticoagulants

## Abstract

**Background:**

Long-term management of chronic conditions, such as atrial fibrillation (AF), require frequent interactions with the healthcare systems. The multinational EUropean Patient Survey in Atrial Fibrillation (EUPS-AF) was conducted to investigate patient satisfaction with AF management in different of five European healthcare systems at a time of changing treatment paradigms for stroke prophylaxis, prior to the advent of newer oral anticoagulants.

**Methods:**

Adults (>18 years) were recruited at random from the total populations of France, Germany, Italy, Spain and the UK using a randomized telephone dialling system. At least 300 respondents per country reporting to have a diagnosis of AF or receiving oral anticoagulation therapy for suspected AF or to have a heart rhythm disturbance completed a structured telephone interview.

**Results:**

Most respondents were satisfied with their treatment for AF over the previous 12 months, with 85.5% (n = 1289) rating their care as good or better. Suboptimal clinical practices, however, were identified in several key areas. Coordination of primary and secondary care and a lack of patient engagement and support were particular issues, especially for those patients likely to have extensive contact with their healthcare system.

**Conclusions:**

In the context of Europe-wide guidelines for management of AF, most patients with AF were satisfied with their care, but for a greater proportion of patients, some aspects are unsatisfactory. Patient-centred surveys, such as the EUPS-AF, are crucial for understanding the factors that contribute to patient satisfaction and compliance with long-term treatment for chronic conditions.

## Background

Atrial fibrillation (AF) is the most common cardiac rhythm disorder, with a prevalence of between 1% and 2% in the general population [1,2]. AF affects approximately 6 million individuals in Europe and is anticipated to double in prevalence over the next 50 years as the population ages [1]. Patients with AF typically require long-term management, including anticoagulation, to prevent debilitating clinical sequelae, such as stroke and heart failure, [1,3] costing European healthcare systems up to €13.5 billion per annum [3].

Due to the complex nature of AF and the associated management pathway, patients typically have extensive contact with healthcare providers, and thus require a high level of communication and engagement to achieve optimal treatment outcomes and satisfaction with treatment. According to the Institute of Medicine’s report in 2001, focusing on the needs of patients should be central to all healthcare provision [4]. Understanding patient attitudes to, and preferences for, treatment of chronic conditions is therefore crucial for optimizing healthcare strategies [5]. However, a survey of almost 10,000 patients in eight countries worldwide has shown that management of patients with chronic conditions is poorly coordinated and that there is a suboptimal level of satisfaction with the standard of care provided [6].

AF serves as a model of how healthcare resources can be organized to optimize long-term treatment of chronic conditions to prevent or delay the need for acute interventions. However, insight into factors that achieve patient satisfaction with treatment of AF is limited, with much of the published information focusing on examining patient attitudes towards anticoagulation therapy, for example, [7,8] rather than on the patient experience as a whole. Moreover, patient surveys are rarely randomized, and thus the results must be interpreted in light of potential sampling bias.

The EUropean Patient Survey in Atrial Fibrillation (EUPS-AF) was conducted to assess patient satisfaction with the management of AF in France, Germany, Italy, Spain, and the UK, and to identify areas in which care could be improved, thereby providing an evidence-based platform for optimizing patient-centred care. The survey was conducted prior to the approval and widespread uptake of novel oral anticoagulants, the direct thrombin and factor Xa inhibitors that have the benefit of not requiring anticoagulation testing. It represents therefore a unique opportunity to establish patient preferences for AF management as we move into a new era of clinical practice.

Here, we use the rigorous Commonwealth Fund Survey methodology, which has been used widely to assess the level of satisfaction with long-term treatment of chronically ill patients, [6,9,10] to highlight areas where healthcare improvements should be targeted to facilitate patient engagement and increase patient satisfaction with AF management.

## Methods

The EUPS-AF was based on a survey developed by the Commonwealth Fund to analyse patient attitudes to, and satisfaction with, treatment for chronic conditions, with focus on access to appropriate healthcare, coordination of services, and safety [6]. In addition to the Commonwealth Fund Survey questions, the EUPS-AF included questions pertinent to patients receiving treatment for AF developed by a steering group comprising clinicians and health economists, in order to better characterize the current population.

The authors declare, that the survey referred to in this manuscript was not involving any biomedical research involving human subjects, and that no such research have been performed nor used in the course of the survey.

### Participants

Structured telephone interviews were carried out to identify individuals older than 18 years who either responded positively to have a diagnosis of AF or where not sure but responded that they were receiving oral anticoagulation therapy for either suspected AF or a heart rhythm disturbance, from a random population of individuals with a telephone landline in France, Germany, Italy, Spain and the UK. The sample was stratified with random sampling conducted within a stratum made up of the five countries. The sample size and power of the survey were pragmatic, consisting of approximately 300 participants per country.

### Survey methodology

After a pilot survey involving 152 respondents conducted in February and March 2011, the questionnaire was adapted minimally to explain more clearly some medical terms associated with AF and to remind respondents to base their answers specifically on their experiences of AF management. The main survey was conducted between 1 May and 18 July 2011. Overall, 1507 individuals were surveyed, including those taking part in the pilot survey.

#### Questionnaire

**Figure 1:**
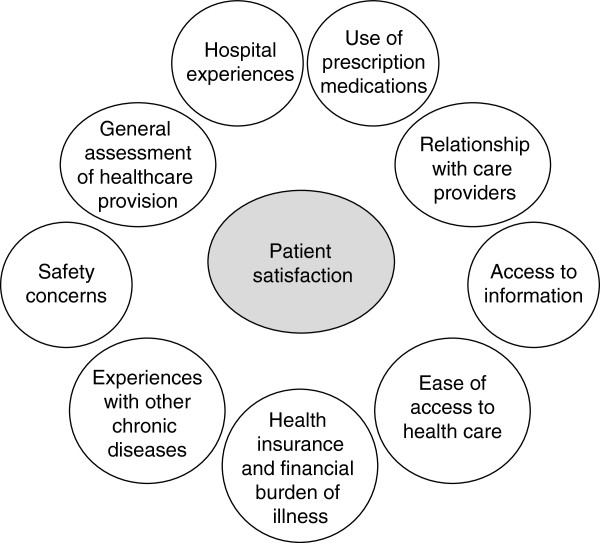
EUropean Patient Survey in Atrial Fibrillation (EUPS-AF) questions encompassed nine domains relating to atrial fibrillation management.

Interviews based on the questionnaire [see Additional file 1], with verified translations into local languages, were conducted by telephone using a computer-assisted telephone interview (CATI) technique [11]. Eligible individuals agreeing to participate in the survey were then asked a series of questions concerning levels of satisfaction with healthcare provision in nine domains (Figure 1). A large amount of data was collected under each domain, and in the current paper, results from various domains are being presented, with the common focus on healthcare improvement, patient engagement and patient satisfaction with AF management.

Participants were not provided with any incentives to participate in the survey. The survey did not require ethical review but, before each interview, respondents were informed about the purpose of data collection and provided their verbal consent to participate in the survey and for the data to be reported. Participants were aware that they could terminate the interview at any point.

### Assessment of stroke risk

The CHADS_2_ (Congestive heart failure, Hypertension, Age, Diabetes, prior Stroke) scoring system calculates the annual risk of stroke based on whether a patient has congestive heart failure, hypertension (blood pressure consistently ≥140/90 mm Hg), is aged at least 75 years or more, has diabetes mellitus, or has a prior history of stroke or transient ischemic attack. Physicians use this information to assess the risk–benefit ratio for administering anticoagulation therapy for stroke prevention in patients with AF [1]. Participants were stratified into those at low, medium, and high risk of stroke (scores of 0, 1–2 or >2, respectively).

### Data analysis

For quantitative parameters, summary statistics, mean ± standard deviation are reported. For qualitative and ordinal parameters, the number of patients within a specific category are presented as absolute and percentage numbers [n (%)], using the total number of patients as denominator (N). Missing values are considered as a valid category ‘no response’.

## Results

### Response rate and participant characteristics

In total, 568,339 telephone numbers were randomly generated, and a total of 340,476 numbers were dialled in order to obtain the minimum of 300 participants with AF per country. Of the 1828 individuals contacted who met the EUPS-AF inclusion criteria, 321 declined to participate in the survey. The average duration of the telephone interviews was 39 minutes (range, 16–151 minutes).

Respondent characteristics are shown in Table 1. Mean age was 70.1 ± 12.5 years (range, was 70.1 ± 12.5 years (range, 68.5–70.8 years) with approximately equal numbers of men and women. Respondent characteristics were generally well matched across countries in terms of age, sex, CHADS_2_ score, and comorbidities.

**Table 1:** Demographic characteristics of the EUropean patient survey in Atrial Fibrillation respondents*

	**France (N = 300)**	**Germany (N = 300)**	**Italy (N = 302)**	**Spain (N = 305)**	**UK (N = 300)**	**Total (N = 1507)**
Mean age, years (SD)	68.5 (13.7)	70.4 (10.5)	70.8 (12.2)	70.8 (13.7)	70.3 (11.9)	70.1 (12.5)
<50 years, n (%)	22 (7.3)	17 (5.7)	19 (6.3)	24 (7.9)	20 (6.7)	102 (6.8)
50– < 65 years, n (%)	91 (30.3)	55 (18.3)	57 (18.9)	54 (17.7)	65 (21.7)	322 (21.4)
≥65 years, n (%)	187 (62.3)	228 (76.0)	226 (74.8)	227 (74.4)	215 (71.7)	1083 (71.9)
Male, n (%)	153 (51.0)	154 (51.3)	152 (50.3)	138 (45.2)	153 (51.0)	750 (49.8)
Receiving oral anticoagulation n (%)^**^						
Yes	215 (71.7)	232 (77.3)	224 (74.2)	253 (83.0)	231 (77.0)	1155 (76.6)
No	54 (18.0)	37 (12.3)	36 (11.9)	24 (7.9)	36 (12.0)	187 (12.4)
No response	6 (2.0)	2 (0.7)	13 (4.3)	5 (1.6)	4 (1.3)	30 (2.0)
Not asked	25 (8.3)	29 (9.7)	29 (9.6)	23 (7.5)	29 (9.7)	135 (9.0)
**Income, n (%)**						
Below average	156 (52.0)	175 (58.3)	169 (56.0)	196 (64.3)	154 (51.3)	850 (56.4)
Average	65 (21.7)	37 (12.3)	90 (29.8)	42 (13.8)	59 (19.7)	293 (19.4)
Above average	50 (16.7)	53 (17.7)	16 (5.3)	23 (7.5)	57 (19.0)	199 (13.2)
No response	29 (9.7)	35 (11.7)	27 (8.9)	34 (14.4)	30 (10.0)	165 (10.9)
**CHADS**_**2**_**score, n (%)**						
0	10 (3.3)	9 (3.0)	10 (3.3)	10 (3.3)	6 (2.0)	45 (3.0)
1–2	74 (24.7)	83 (27.7)	68 (22.5)	70 (23.0)	82 (27.3)	377 (25.0)
>2	216 (72.0)	208 (69.3)	224 (74.2)	225 (73.8)	212 (70.7)	1085 (72.0)
**Number of current medications**						
Mean (SD)	5.7 (4.1)	5.4 (4.0)	5.4 (3.3)	6.0 (3.9)	6.1 (4.1)	5.7 (3.9)
>10 medications, n (%)	46 (15.3)	26 (8.7)	34 (11.3)	45 (14.8)	48 (16.0)	199 (13.2)
**Where respondents live, n (%)**						
City/large town	99 (33.0)	76 (25.3)	45 (14.9)	138 (45.2)	105 (35.0)	463 (30.7)
Small town	87 (29.0)	92 (30.7)	90 (29.8)	28 (9.2)	89 (29.7)	386 (25.6)
Village/rural location	113 (37.7)	130 (43.3)	165 (54.6)	138 (45.2)	105 (35.0)	651 (43.2)
No response	1 (0.33)	2 (0.67)	2 (0.66)	1 (0.33)	1 (0.33)	7 (0.46)
**Comorbidities, n (%)**						
Mean	2.1	2.1	2.2	2.3	2.3	2.2
Hypertension	152 (50.7)	208 (69.3)	197 (65.2)	180 (59.0)	185 (61.7)	922 (61.2)
Heart disease	165 (55.0)	167 (55.7)	130 (43.0)	141 (46.2)	135 (45.0)	738 (49.0)
Diabetes	60 (20.0)	78 (26.0)	60 (19.9)	68 (22.3)	50 (16.7)	316 (21.0)
Arthritis	69 (23.0)	47 (15.7)	127 (42.1)	103 (33.8)	144 (48.0)	490 (32.5)
Asthma, COPD, or other chronic lung disease	55 (18.3)	53 (17.7)	48 (15.9)	63 (20.7)	49 (16.3)	268 (17.8)
Depression, anxiety, or other mental health problem	90 (30.0)	47 (15.7)	75 (24.8)	126 (41.3)	77 (25.7)	415 (27.5)
Cancer	39 (13.0)	43 (14.3)	31 (10.3)	34 (11.1)	40 (13.3)	187 (12.4)
Stroke	78 (26.0)	31 (10.3)	59 (19.5)	63 (20.7)	95 (31.7)	326 (21.6)

^*^These data were patient-reported and were not confirmed by a treating clinician.

**This question was added to the survey during the pilot phase of the survey and therefore not all respondents were asked.

AF = atrial fibrillation; CHADS = congestive heart failure, hypertension, age, diabetes, prior stroke; COPD = chronic obstructive pulmonary disease; SD = standard deviation.

### Patient opinion on their healthcare system

Participants were asked to provide an opinion on their national healthcare system by selecting one of the following statements: “on the whole the system works pretty well, and only minor changes are necessary to make it work better”, “there are some good things in our healthcare system, but fundamental changes are needed to make it work better”, “our healthcare system has so much wrong with it that we need to completely rebuild it”. Overall, 44.7% (n = 673) thought that their national healthcare system worked ‘pretty well’ and that only minor changes were necessary to make it work better. Patients in the UK were more likely to declare this statement to be true (54.7% positive responses [n = 164]) than patients in France, Germany, Italy, or Spain (range, 37.1 – 46.9% [n = 112–143]). Patients in the UK were also less likely to state that their healthcare system had so much wrong with it that it needed rebuilding completely (3.7% [n = 11]) in comparison to the other four surveyed countries (range, 11.0 – 17.5% [n = 33–53]).

### Patient satisfaction with quality of care

**Figure 2:**
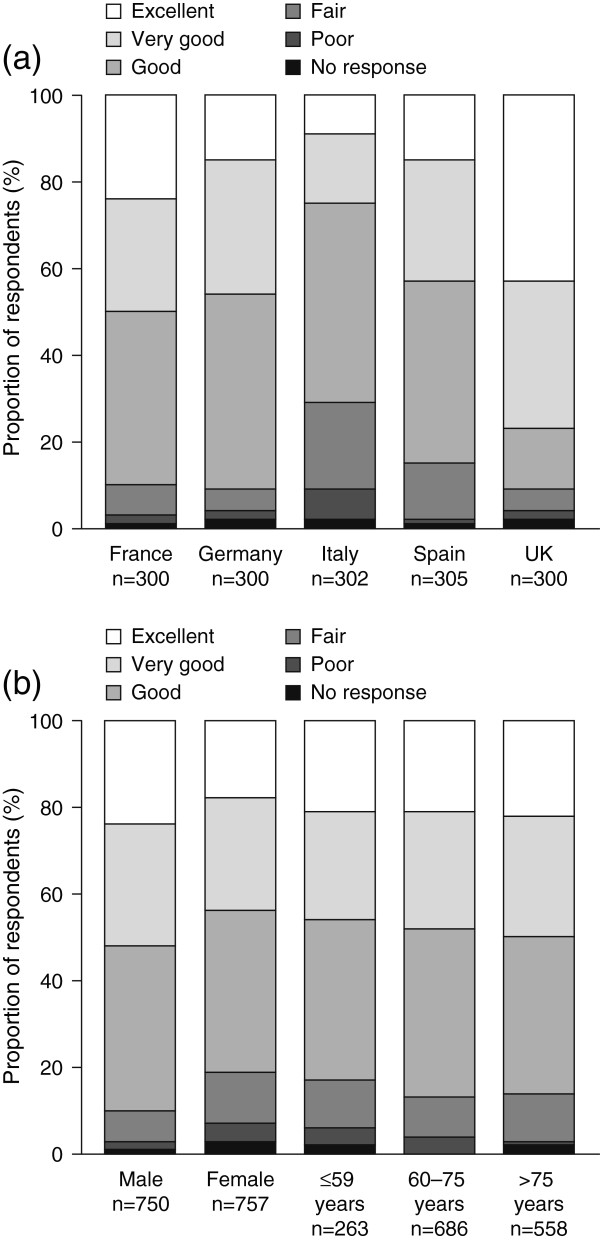
**Patient rating of quality of medical care. (a)** Patient rating of quality of medical care received during the previous 12 months and **(b)** overall rating of quality of care by age and sex. Survey question: Overall, how do you rate the quality of medical care that you have received in the past 12 months?

Participants were asked to rate the quality of care they had received in the previous 12 months using a five-point Likert scale (excellent, very good, good, fair or poor). Overall, 85.5% of respondents (n = 1289) rated the quality of care they had received in the previous 12 months as good, very good, or excellent (range, 71.5 – 91.7% [n = 216 – 275]) (Figure 2a). Respondents in Germany, the UK, and France were more likely to report care that was good, very good, or excellent (Germany, 90.7% [n = 272] of participants; UK, 91.7% [n = 275]; France, 89.7% [n = 269]) than were individuals in Italy or Spain (71.5% [n = 216] and 84.3% [n = 257], respectively). Dissatisfaction with medical care was higher in Italy (26.8% [n = 81] rated care as fair or poor) than in the other four countries surveyed (range, 6.7–14.8% [n = 20–45]).

The overall rating of quality of care did not differ substantially between men and women, or between different age groups (Figure 2b).

### Coordination of care

The proportion of patients who had seen a specialist during the previous 2 years varied across countries. In France, patients were more likely to have seen a specialist than in the other countries surveyed (89.7% [n = 269] vs. 68.3 – 78.3% [n = 205–238]). Moreover, waiting times to see a specialist varied widely, from an average of 4.0 weeks in Germany to 11.9 weeks in Spain; 40.8% (n = 97) of Spanish respondents had to wait for more than 8 weeks to see a specialist, a higher proportion than in the other countries surveyed (range, 16.7–25.9% [n = 43–54]). Patients with the highest CHADS_2_ score (score 5–6) had to wait an average of 7.8 weeks to see a specialist, longer than patients with lower CHADS_2_ scores (range, 4.4–7.2 weeks).

Patients with high CHADS_2_ scores (score 5–6) reported the most problems with coordination of care. More patients in this group felt that they had been recommended treatment that had little health benefit (22%) than did patients with lower scores (CHADS_2_ score, 0–4; range, 15–20%). A greater proportion of patients (16%) with high CHADS_2_ scores thought that medical tests had been performed unnecessarily, compared with lower CHADS_2_ scores (range, 9–13%).

**Figure 3:**
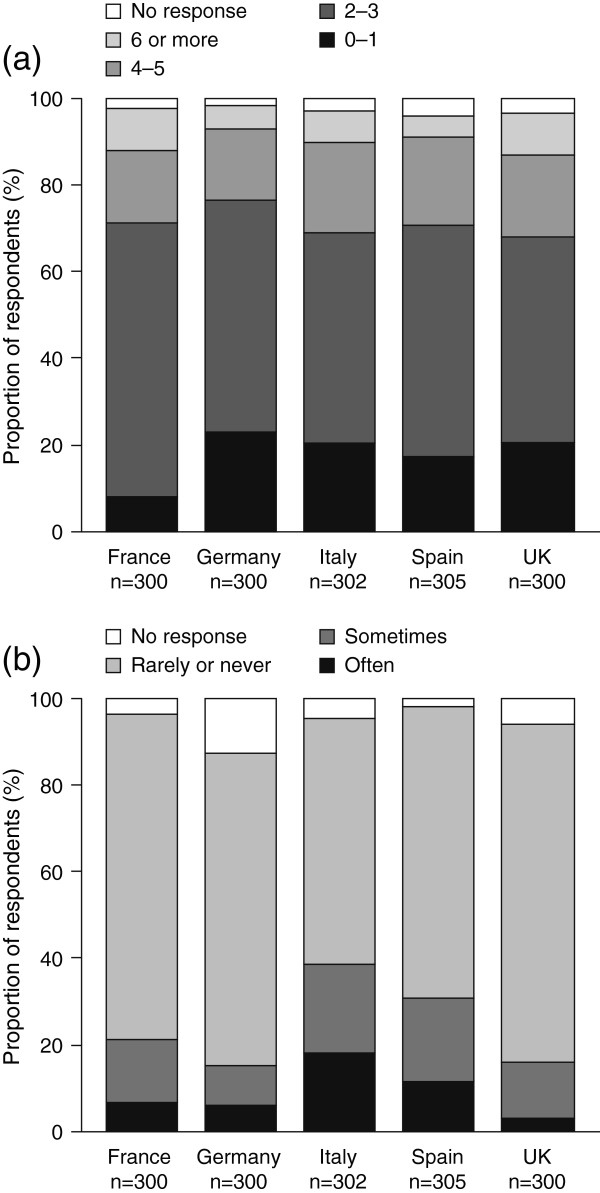
**Medical care organization. (a)** Number of general practitioners or specialists seen by patients during the previous 2 years and **(b)** proportion of patients who believed that their time had been wasted due to poorly organized care during the previous 2 years. Survey questions: **(a)** How many different doctors have you seen in the past 2 years, including your regular doctor (the doctor you rely on most for your care) and any specialist doctors or consultants? **(b)** In the past 2 years, how often did you feel your time was wasted because your medical care was poorly organized?

Overall, 79.3% of respondents (n = 1195) had seen more than one doctor during the previous 2 years for the same condition. This is interpreted as a marker for potential discontinuity in care (Figure 3a). There were differences between countries in how informed patients felt that their regular doctor was about the care the patient received from the specialist they had been seeing. In France, only 4.0% (n = 10) of patients felt that their regular doctor was not informed about their specialists’ input to their condition, while in Italy and Spain this figure was higher (28.3% [n = 41] and 26.7% [n = 50], respectively). Many patients felt that their time had often, or occasionally, been wasted because care was poorly organized (Figure 3b). In Italy, 18.2% (n = 55) of respondents felt that their time had often been wasted due to poor coordination, a greater proportion than in the other countries surveyed (range, 3.0–11.5%, [n = 9–35]). Women were more likely than men to feel that their time had been wasted (10.8% [n = 82] vs. 7.3% [n = 55]). Neither income nor age affected the perception of wasted time.

### Current medications

#### Prescription medication use

**Figure 4:**
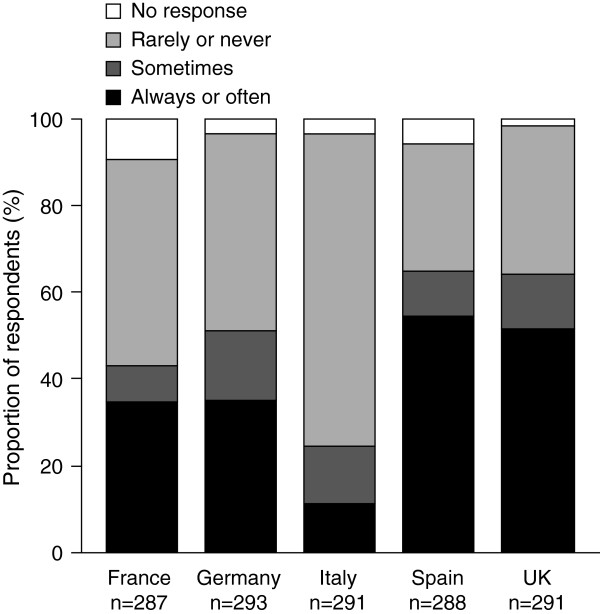
**Frequency with which medications were reviewed by doctors or pharmacists during the previous 2 years.** Survey question: In the past 2 years, how often have any of your doctors or your pharmacists reviewed and discussed all the different medications you are using, including medicines prescribed by other doctors?

On average, respondents were taking approximately six prescription medications on a regular basis, and 13.2% (n = 199) of patients were taking 10 or more prescription medications (range, 8.7–16.0% [n = 26–48]) (Figure 4). In total, 80.7% (n = 918) of patients expressed a preference for taking anticoagulation medication once daily compared with only 7.6% (n = 87) who preferred a twice-daily regimen.

Differences in the extent to which doctors or pharmacists engaged with patients to review their medication were observed between countries (Figure 4). A greater proportion of respondents in Italy rarely or never had their medication reviewed compared with the other countries surveyed (71.8% [n = 209] vs. 29.2–47.4%, [n = 84–136]). Moreover, a greater proportion of patients in Spain always had their medication reviewed (41.0%, n = 118) than in the other four European countries (range, 4.8–27.5% [n = 14–80]).

The proportion of patients who had been informed of the potential side effects of their medication was low in all the countries surveyed (mean, 14.6% [n = 212] of respondents; range, 8.9–30.3% [n = 26–87]). A greater proportion of patients in France had been informed about potential medication side effects when filling a prescription (30.3%, n = 87) compared with their European counterparts (range, 8.9–11.7%) [n = 26–35].

#### Anticoagulation testing

**Figure 5:**
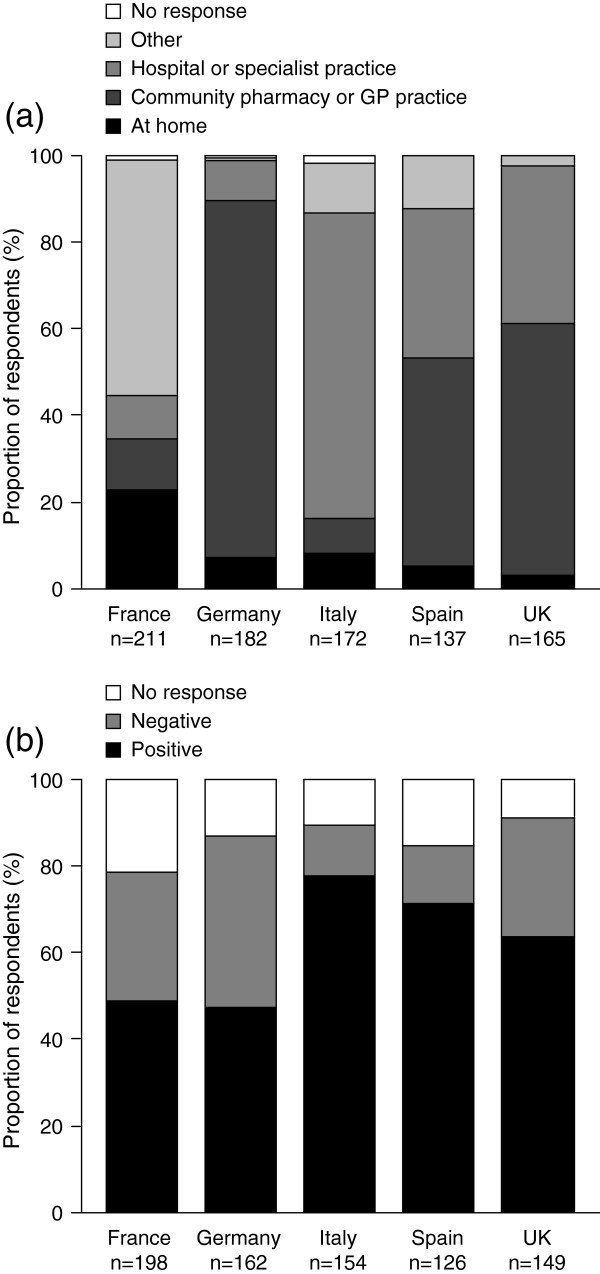
**Anticoagulation testing. (a)** Settings for anticoagulation testing and **(b)** attitudes to no longer needing regular anticoagulation testing. Survey questions: **(a)** Where is your PT/INR monitored? **(b)** What do you think about the possibility of no longer having the need to get coagulation test done like PT/INR? GP = general practitioner; INR = international normalized ratio; PT = prothrombin time.

Differences in approaches towards anticoagulation testing to determine patients’ international normalized ratio (INR) values were observed among countries (Figure 5). A greater proportion of French patients with AF received regular anticoagulation testing (71.5%, n = 211) than was reported for other countries (range, 46.1–60.9% [n = 137–182]), with more than half of tests taking place in a local, specialized laboratory. Conversely, Italian respondents were not only less likely to receive regular anticoagulation testing than their French counterparts (57.3%, [n = 172] vs. 71.5%, [n = 211]), but were more likely to have to travel to a hospital for testing (63.4%, n = 109) than respondents from any of the other European countries surveyed (range, 2.2–33.3% [n = 4–55]).

Healthcare systems differed in the setting in which patients received anticoagulation testing (Figure 5a). In Germany, more respondents (82.4%, n = 150) received anticoagulation testing at their local doctor’s surgery than in the other European countries (range, 7.6–53.3% [n = 13–88]). Overall, just 2.5% of respondents (n = 22) self-administered their anticoagulation test at home, prevalent only in Germany and Italy, 5.5% (n = 10) and 5.2% (n = 9), respectively. Patients in France were more likely to have their anticoagulation test performed at home by a district nurse (21.3% [n = 45]) than in the other European countries (range, 1.6–5.1% [n = 3–7]).

Italian respondents were the most positive about the benefit of reduced anticoagulation testing (77.9% [n = 120] positive responses) (Figure 5b). Individuals in France and Germany were less positive about a reduction in monitoring requirements (49.0% [n = 97] and 47.5% [n = 77] positive responses, respectively) than patients in Italy, Spain, and the UK (range, 63.8–77.9% [n = 95–120]).

Across all countries surveyed, 28.0% (n = 134) of respondents would favour reduced anticoagulation testing because of the time saved, while 29.0% (n = 139) cited the reason as a reduction in the need to travel regularly to an appropriate testing centre. Of those respondents not in favour of reduced testing (mean, 25.2% [n = 199]; range, 11.7–39.5% [n = 18–64]), many were either concerned about the uncertainty of not knowing their anticoagulation status (mean, 31.7% [n = 63]; range, 17.6–45.3% [n = 3–29]) or felt that they needed to know their INR value (mean, 41.7% [n = 83]; range, 22.2–53.7% [4–22]).

## Discussion

The multinational EUPS-AF highlights international differences in the management of patients with AF despite Europe-wide treatment guidelines, in particular widely varying service delivery of therapies such as anticoagulation. Although the majority of patients are satisfied with their care, some individuals experience poorly coordinated care and lack of engagement and support when receiving treatment for this complex, chronic and burdensome condition.

### Factors affecting patient satisfaction

Management of patients with AF, many of whom has one or more comorbidities and frequently receives several prescriptions, is often complex and burdensome, leaving some patients vulnerable to miscommunication, lack of engagement, and reduced coordination of care. Medication reviews, education and access to self-testing therapies, providing some autonomy to the patients, is variable and limited. In this regard, novel oral anticoagulants that do not require patients to undertake regular anticoagulation monitoring and dose adjustment may improve patient satisfaction with management. Differences in attitudes towards anticoagulation testing among countries, however, indicate that doctors should carefully consider patients’ anticoagulation preferences. In France and Germany, many of those patients not in favour of transitioning to new oral anticoagulants cited the peace of mind that regular consultations for anticoagulation testing with healthcare professionals provide, highlighting that less contact with doctors is not always a desirable outcome for patients. Moreover, it is tempting to speculate that the relative convenience of anticoagulation testing in France and Germany, in a local laboratory or doctors’ practice, results in less enthusiasm towards the reduced need for regular anticoagulation testing with novel anticoagulants. In the UK, Spain, and Italy, where testing is less convenient than in France or Germany, and therefore poses a greater burden to patients, a higher proportion of respondents were in favour of reduced monitoring. Thus the EUPS-AF results suggest that the convenience and location of anticoagulation testing may contribute to patients’ attitudes towards regular monitoring.

#### Access to healthcare

Where patients live, and how far they have to travel for consultation and monitoring, is also likely to contribute substantially to their satisfaction with AF management. Respondents in Italy were more likely to report to live in rural areas than respondents from other countries, and therefore also more likely to have to travel to see a specialist in hospital to receive their care. The impact of such a patient burden on their attitude to, and satisfaction with, their care should not be underestimated and reflects an important economic consideration. Many patients with AF are elderly and thus may have difficulty travelling to hospital clinics to have their anticoagulation status monitored on a regular basis.

#### Coordination of care

Receiving healthcare through multiple sources can increase the risk of reduced coordination of care; for example, through miscommunication between primary and secondary care. Almost one-third of patients in Italy and Spain felt that their regular doctor was not well informed about the care they had received from specialists, compared with only 4% of patients in France. Waiting times were longest in the UK and Spain, where access to secondary care has been a long-standing issue for policymakers, while patients in Germany experienced the shortest waiting times to see a specialist. Despite almost half of respondents from Spain more often reviewing their medication with their doctor, they were no better informed about the potential side effects of their medications than patients who reviewed their medication less often with health care professionals. Moreover, the present survey indicates that there is still a need for education for patients about their anticoagulation treatment, especially referring to the proportion of patients with high CHADS_2_ scores, feeling that they had been recommended treatment with little health benefit and that medical tests had been performed unnecessarily.

### EUPS-AF results in context

Results from the EUPS-AF confirm findings from the Commonwealth Fund Survey that improvements in healthcare systems need to be implemented to increase patient satisfaction and compliance with long-term treatment of chronic conditions, [6,10] and to comply with the guidelines on AF management [1]. While both the EUPS-AF and Commonwealth Fund Survey demonstrated broad differences in levels of satisfaction across countries, it is particularly notable that both surveys identified that levels of satisfaction were higher in the UK than in other countries. This may reflect initiatives in the UK in the past 5 years to improve delivery and coordination of healthcare to patients with chronic conditions. Differences between countries may also be due to cultural differences. Another factor to be taken into account when interpreting the results of EUPS-AF is the effect on the healthcare systems that the economic crisis may have [12-14].

Initiatives from patient support groups, such as the Atrial Fibrillation Association, [15] and recent European guidelines on the prevention of cardiovascular disease, [16] further highlight an increasing awareness of patient-centred care for cardiac conditions. Moving towards healthcare pathways within which patients are able to exert some control over their management is a key goal in increasing satisfaction with treatment, and thus outcomes, in patients with a wide range of conditions [17,18].

A lack of patient engagement in managing stroke prophylaxis has previously been highlighted as an issue by Aliot and colleagues, who suggested that patients should be allowed to make informed decisions about their treatment [19]. Evidence shows that patients with AF appear to place more value on avoiding stroke and subsequent hospitalization than their physicians, who appear more concerned with the risk of haemorrhage, [19,20] further emphasizes the need for patients to be involved in treatment decisions. Moreover, MacLean and colleagues have recently observed that previous patient experience substantially impacts on treatment preferences [7]. A systematic review of stroke prophylaxis in patients with AF revealed substantial heterogeneity in patient treatment preferences, and the review emphasized that patients should be assessed and treated on an individual basis [7]. Engagement and communication with patients, including providing sufficient information and support for them to participate in management decisions, should be a key consideration for healthcare professionals.

To the best of our knowledge, the EUPS-AF represents the largest ever survey of patient satisfaction with AF management. Unlike previous surveys, which have generally focused on patient and physician attitudes towards anticoagulation alone, the EUPS-AF survey highlights patient attitudes towards, and satisfaction with, AF management as a whole, in the context of national healthcare systems. Furthermore, the countries surveyed were chosen to represent a diverse range of healthcare systems from those that have adopted the unified guidance framework for AF management [1].

The EUPS-AF demonstrates that it is possible to adapt the rigorous Commonwealth Fund methodology to assess differences in patient satisfaction with treatment for a specific chronic condition. Randomized telephone dialling reduced the selection bias often associated with patient surveys, and ensured that a representative range of patients with AF was included.

The survey does have a number of limitations. Despite the randomized selection protocol, there is potential for selection bias given that only those who were willing to take the time to respond to the calls were included in the survey. Moreover, the computer-assisted telephone interviewing technique is inherently biased towards those individuals with static telephone lines, excluding individuals without a permanent landline. Regional variance in telephone access amongst countries was not accounted for. The biases of a primary care only population or of patients with reasonable mobility to undertake research were however minimized by randomly detecting these patients by telephone. The sample size was pragmatic, and the precision of patient characteristics and power to detect level of difference between countries were not taken into account when planning the sample size. For country differences, no weighting of the results in relation to difference in prevalence between countries has been used. The survey did not address surgical interventions for the management of AF or explore the influence of alternative rate control therapies, such as the ‘pill-in-a-pocket’ strategy, on patient satisfaction. Source data relied fully on patient volunteered responses and outcomes, and was neither verified with their treating physicians, nor weighted for differences in living environment, age or type of AF (paroxysmal, persistent or permanent). Since the time of data collection, the recommendation of scoring of the European Society of Cardiology guidelines on the management of AF had changed from CHADS^2^ to CHADS^2^VASc and this was not taken into account in the current data analysis [21]. CHADS^2^VASc gives a better stratification of low-risk patients.

## Conclusions

Patient satisfaction with treatment is a key component of the successful management of chronic conditions such as AF. Management of AF patients in real-world practice shows considerable variation and is often not consistent with current recommendations, and the survey supports the need to include aspects of patient-centred care in treatment guidelines. The survey also identified key areas where healthcare systems can improve in order to optimize patient satisfaction with AF management, including increasing patient education and engagement, service delivery models and coordination of care.

Moreover, the survey highlights satisfaction with AF management during the key period before novel anticoagulants enter regular clinical practice, thus providing a snapshot of AF management at a time of changing treatment paradigms. Further research will be required to assess the impact of the introduction of novel anticoagulants on satisfaction of patients with AF management and to identify the best method of adopting a patient perspective into AF management strategies.

## Abbreviations

AF: Atrial Fibrillation; CATI: Computer-assisted telephone interview; CHADS2 score: Heart failure, hypertension, age, diabetes, prior stroke or TIA double; EUPS-AF: European patient survey in atrial fibrillation; GP: General practitioner; INR: International normalized ratio; PT: Prothrombin time.

## Competing interests

AB has received speaker and advisory board honoraria from multiple device and pharmaceutical partners, including Daiichi Sankyo Europe GmbH, Bayer and Boehringer Ingelheim. He is a director of AMORE Health Ltd and works within the NHS and also for Health Smart Ltd. He is R & D lead at Barnet & Chase Farm NHS Trust and participates in clinical trials sponsored by a variety of companies. AS is an employee of Daiichi Sankyo Europe GmbH. TM has previously received consulting fees from Daiichi Sankyo Europe GmbH. WG, PB and TL declare no conflicts of interest. EF is an employee of Harris Interactive. AMSO was an employee of Daiichi Sankyo Europe GmbH when the survey was conducted. JLZ has received speaker and advisory board honoraria from Daiichi Sankyo Europe GmbH.

## Authors’ contributions

AMSO and AS initiated and led the survey. EF was responsible for data collection and compilation. WG and JLZ contributed to survey and questionnaire design, and, together with AB, BP, TM and TL, analysed the data results from professional and country specific expertise. AB chaired the collaborating between authors. All authors contributed to drafting, review and revise the manuscript. All authors read and approved the final manuscript.

## Pre-publication history

The pre-publication history for this paper can be accessed here:

http://www.biomedcentral.com/1471-2261/13/108/prepub

## Supplementary Material

Additional file 1 The EUropean Patient Survey in Atrial Fibrillation (EUPS-AF) Questionnaire. Click here for file
