# Supplementary material for: Patient perspective on the management of atrial fibrillation in five European countries
Source: BMC Cardiovasc Disord. 2013 Dec 1;13:108. doi: 10.1186/1471-2261-13-108 (PMC4219387; doi:10.1186/1471-2261-13-108)
Supplement: Additional file 1 — The EUropean Patient Survey in Atrial Fibrillation (EUPS-AF) Questionnaire. [file 1471-2261-13-108-S1.pdf]

The EUropean Patient Survey in Atrial Fibrillation (EUPS-AF) Questionnaire

Original questionnaire: Schoen C, Osborn R, How SK, Doty MM, Peugh J. *In Chronic Condition: Experiences Of Patients With Complex Health Care Needs, In Eight Countries, 2008.* Health Aff (Millwood). 2009 Jan-Feb;28(1):w1-16.

Information to the reader: During the interview the interviewer reminder the patient several times to have an atrial fibrillation perspective in mind when answering the questions (see below interviewer information). Throughout interview interviewer instructions were used for the interviewer to explain questions and expressions to the patients.

Contents

SECTION 1: PRELOADED SAMPLE VARIABLES 1
SECTION 2: INTRODUCTION AND SCREENING 2
SECTION 3: OVERVIEW OF HEALTH SYSTEM 4
SECTION 4: ACCESS TO HEALTH CARE 4
SECTION 5: RELATIONSHIP WITH REGULAR DOCTOR 5
SECTION 6: COORDINATION OF CARE 7
SECTION 7: EXPERIENCE WITH SPECIALISTS 8
SECTION 8: PRESCRIPTION MEDICATION USE 9
SECTION 9: MEDICAL ERRORS/SAFETY ISSUES 11
SECTION 10: EXPERIENCES WITH CARE IN HOSPITAL 13
SECTION 11: EMERGENCY ROOM USE 14
SECTION 12: CHRONIC ILLNESS CARE 14
SECTION 13: RATING OF OVERALL CARE 18
SECTION 14: HEALTH CARE COVERAGE 20
SECTION 15: OUT OF POCKET COSTS 21
SECTION 16: DEMOGRAPHICS 22

Interviewer information: We are primarily interested in the patients' experiences from their perspective as atrial fibrillation patients. Whenever they ask you if you are asking from a general or from an atrial fibrillation perspective you can confirm that it is from an atrial fibrillation perspective.

SECTION 1: PRELOADED SAMPLE VARIABLES

BASE: ALL RESPONDENTS

Q1_ 1 Country

1. France
2. Germany
3. Italy
4. Spain
5. The United Kingdom

SECTION 2: INTRODUCTION AND SCREENING

BASE: ALL RESPONDENTS

Q2_1 I would like to speak to the person in the household who is 18 or older. Would you be that person?

1. Continue
2. Not available – call back
8. Not sure
9. Decline to answer

BASE: ALL CONTINUING RESPONDENTS (Q2_1/1)

Q2_2 First, what is your year of birth?

[RANGE: 1906-2010, 9998 = not sure, 9999=decline to answer]

BASE: ALL CONTINUING RESPONDENTS (Q2_1/1)

Q2_3 Age categories

1. 1-17 (2010-1994)
2. 18-24 (1993-1987)
3. 25-34 (1986-1977)
4. 35-49 (1976 - 1962)
5. 50-64 (1961-1947)
6. 65+ (1946-1906)
8 Not sure
9. Decline to answer

BASE: ALL RESPONDENTS WHO ARE Q2_3/2-6

Q2_4 Respondent sex

1. Male
2. Female

BASE: ALL RESPONDENTS WHO ARE Q2_3/2-6

Q2_5 Are you diagnosed with atrial fibrillation?

1. Yes
2. No
8. Not sure
9. Decline to answer

BASE: ALL RESPONDENTS

Q2_5_1 Do you take oral anticoagulation?

1. Yes
2. No
8. Not sure
9. Decline to answer

BASE: ALL RESPONDENTS WHO ARE NOT SURE Q2_5/8 AND TAKING ANTICOAGULANTS Q2_5_1/1

Q2_5_2 Why are you taking anticoagulants?

A Atrial fibrillation
B Heart rhythm disturbance
C Atrial flutter
D Artificial heart valve
E Thrombosis
F Others ______________________________

BASE: ALL RESPONDENTS

Q2_6 In general, how would you describe your own health?

1. Excellent
2. Very good
3. Good
4. Fair
5. Poor
8. Not sure
9. Decline to answer

BASE: ALL RESPONDENTS

Q2_7 Were you hospitalized in the past 2 years? [other than for a normal, uncomplicated delivery of a baby]?

1. Yes
2. No
8. Not sure
9. Decline to answer

BASE: ALL RESPONDENTS

Q2_8

1. Qualified respondent
2. Not qualified respondent; thank and determine

BASE: ALL QUALIFIED RESPONDENTS

Q2_9

1. France
2. Germany
3. Italy
4. Spain
5. The United Kingdom

SECTION 3: OVERVIEW OF HEALTH SYSTEM

BASE: ALL RESPONDENTS

Q3_1 Which of the following statements comes closest to expressing your overall view of the health care system in this country?

1. On the whole, the system works pretty well and only minor changes are necessary to make it work better.
2. There are some good things in our health care system, but fundamental changes are needed to make it work better.
3. Our health care system has so much wrong with it that we need to completely rebuild it.
8. Not sure
9. Decline to answer

SECTION 4: ACCESS TO HEALTH CARE

BASE: ALL RESPONDENTS

Q4_1 Was there a time when you… because of the cost in the past 2 years?

A Did not fill a prescription for medicine or skipped doses
B Had a specific medical problem but did not visit a doctor
C Skipped or did not get a medical test, treatment, or follow-up that was recommended by a doctor

1. Yes
2. No
3. Not applicable
8. Not sure
9. Decline to answer

BASE: ALL RESPONDENTS

Q4_2 Last time when you needed medical care in the evening, on a weekend or on a holiday, how easy or difficult was it to get care without going to the hospital emergency department? Was it…?

1. Very easy
2. Somewhat easy
3. Somewhat difficult
4. Very difficult
5. Never needed care in the evenings, weekends, or holidays
8. Not sure
9. Decline to answer

BASE: ALL RESPONDENTS

Q4_3 Last time you were sick or needed medical attention, how quickly could you get an appointment to see a doctor? Please do not include a visit to the hospital emergency department. Did you get an appointment…

1. On the same day
2. The next day
3. In 2 to 3 days
4. In 4 to 5 days
5. In 6 to 7 days
6. After more than a week
7. Never able to get an appointment
8. Not sure
9. Decline to answer
10. Not applicable [If patient only goes to ER or hospital]

BASE: ALL RESPONDENTS

Q4_4 Have you called a help line for medical or health advice in the past 2 years?

1. Yes
2. No
8. Not sure
9. Decline to answer

BASE:ALL RESPONDENTS WHO USED HELP LINE (Q4_4/1)

Q4_5 Were you able to get the advice or information you needed?

1. Yes, completely
2. Yes, to some extent
3 No
8. Not sure
9. Decline to answer

SECTION 5: RELATIONSHIP WITH REGULAR DOCTOR

BASE: ALL RESPONDENTS

Q5_1 Is there one doctor you usually go to for your medical care?

1. Yes, have a “regular doctor”
2. Yes, but have more than “regular doctor”
3. No, do not have a “regular doctor”
8. Not sure
9. Decline to answer

BASE: ALL RESPONDENTS WHO HAVE A REGULAR DOCTOR Q5_1/1

Q5_1_1 Is your "regular doctor"…?

1. A general practitioner
2. A specialist
8. Not sure
9. Decline to answer

BASE: ALL RESPONDENTS WITH NO REGULAR DOCTOR OR NOT SURE OR DECLINE (Q5_1/3,8,9)

Q5_2 Is there one doctor's group, health center, or clinic you usually go to for most of your medical care? Please do not include the hospital emergency department.

1. Yes, have a usual place for medical care
2. No, do not have a usual place for medical care
8. Not sure
9. Decline to answer

BASE: ALL RESPONDENTS WHO HAVE A REGULAR DOCTOR / PLACE (Q5_1/1 OR Q5_2/1 OR Q5_1/2 AND Q5≠1)

Q5_3 How long have you been seeing this doctor/going to this place for your medical care? [The doctor that they rely on most for their care]

1. Less than 1 year
2. 1 to less than 3 years
3. 3 to less than 5 years
4. 5 years or more
8. Not sure
9. Decline to answer

BASE: ALL RESPONDENTS WITH A REGULAR DOCTOR/GP OR A REGULAR PLACE (Q5_1/1 OR Q5_2/1)

Q5_4 How easy or difficult is it to contact your doctor's practice during regular practice hours by telephone about a health problem? Would you say it is …?

1. Very easy
2. Somewhat easy
3. Somewhat difficult
4. Very difficult
6. Never tried to contact by telephone
8. Not sure
9. Decline to answer

BASE: ALL RESPONDENTS WITH A REGULAR DOCTOR OR PLACE (Q5_1/1 OR Q5_2/1)

Q5_5 When you need care or treatment, how often does your regular doctor…?

A Know important information about your medical history
B Encourage you to ask questions
C Tell you about treatment options and involve you in decisions about the best treatment for you
D Give you clear instructions about symptoms to watch for and when to seek further care or treatment

1. Always
2. Often
3. Sometimes
4. Rarely or never
5. Not applicable
8. Not sure
9. Decline to answer

BASE: ALL RESPONDENTS WHO HAVE A REGULAR DOCTOR OR PLACE (Q5_1/1 OR Q5_2/1)

Q5_6 How often does your regular doctor or someone in your doctor's practice help coordinate or arrange the care you receive from other doctors and places, such as make appointments with a specialist?

1. Always
2. Often
3. Sometimes
4. Rarely or never
5. Not applicable
8. Not sure
9. Decline to answer

SECTION 6: COORDINATION OF CARE

BASE: ALL RESPONDENTS

Q6_1 In the past 2 years, when getting care for a medical problem, was there ever a time when…?

A. Test results, medical records, or reasons for referrals were not available at the time of your scheduled doctor's appointment
B. Doctors ordered a medical test that you felt was unnecessary because the test had already been done
C. Doctors recommended treatment that you thought had little or no health benefit

1. Yes
2. No
3 Not applicable
8. Not sure
9. Decline to answer

BASE: ALL RESPONDENTS

Q6_2 In the past 2 years, how often did you feel your time was wasted because your medical care was poorly organized?

1. Often
2. Sometimes
3. Rarely
4. Never
5. Not applicable
8. Not sure
9. Decline to answer

SECTION 7: EXPERIENCE WITH SPECIALISTS

BASE: ALL RESPONDENTS

Q7_1 Have you seen or needed to see any specialist doctors (or consultants) in the past 2 years?

1. Yes
2. No
8. Not sure
9. Decline to answer

BASE: ALL RESPONDENTS

Q7_2 How many different doctors have you seen in the past 2 years, including your regular doctor (the doctor you rely on most for your care) and any specialist doctors or consultants?

[Range: 0-96, 98=not sure, 99=decline to answer]

BASE: ALL RESPONDENTS WHO SAW OR NEEDED TO SEE A SPECIALIST (Q7_1/1)

Q7_3 After learning you needed to see a specialist or consultant, how many days, weeks or months did you have to wait for an appointment?

Q7_3.1	0	No waiting period
Q7_3.2	1	Days
Q7_3.3	2	Weeks
Q7_3.4	3	Months
Q7_3.5	4	Years
Q7_3.8	8	Not sure
Q7_3.9	9	Decline to answer

BASE: ALL RESPONDENTS WHO SAW OR NEEDED TO SEE A SPECIALIST (Q7_1/1)

Q7_4 When you saw the specialist or consultant, did he or she have information about your medical history?

1. Yes
2. No
8. Not sure
9. Decline to answer

BASE: ALL RESPONDENTS WITH REGULAR DOCTOR AND SEEN A SPECIALIST (Q5_1/1 &Q7_1/1)

Q7_5 After you saw the specialist or consultant, did your regular doctor seem informed and up-to-date about the care you got from the specialist/consultant?

1. Yes
2. No
3. Did not see regular doctor/general practitioner since seeing /consultant
8. Not sure
9. Decline to answer

SECTION 8: PRESCRIPTION MEDICATION USE

BASE: ALL RESPONDENTS

Q8_1 How many different prescription medications are you taking on a regular or ongoing basis?

[Range: 0-96, 98=not sure, 99=decline to answer]

BASE: ALL RESPONDENTS

Q8_2 What kind of medication do you receive specifically for your atrial fibrillation treatment?

____

BASE: ALL RESPONDENTS WHO DID NOT ANSWER Q8_2.1 1/2/3/4/5/6/7/8/9/10 or 11

Q8_2.2 Do you receive one of the following medications for atrial fibrillation treatment?

A Warfarin
B Aspirin
C Coumadin
D Dabigatran [Pradaxa]
E Phenprocoumon [Marcumar and Falithrom]
F Acenocoumarol [Sintrom and Sinthrome]
G Other_____________________________
H Combinations of any of the above
I Phenindione
J Fuindione
K Clopidogrel

1. Yes
2. No
8. Not sure
9. Decline to answer

BASE: ALL RESPONDENTS WHO ARE TAKING MEDICATIONS FOR ATRIAL FIBRILLATION (IF Q2_5_1 =1)

Q8_2.3 Would you prefer to take your anticoagulation medication for atrial fibrillation…?

1. Once daily
2. Twice daily
8. Not sure
9. Decline to answer

BASE: ALL RESPONDENTS WHO ARE TAKING MEDICATIONS FOR ATRIAL FIBRILLATION (IF Q8_2.3_1 or Q8_2.3_2)

8_2.3.1 Why?

____

BASE: ALL RESPONDENTS WHO ARE TAKING MEDICATIONS FOR ATRIAL FIBRILLATION (IF Q2_5_1 =1)

Q8_2.4 What do you think of the possibility of no longer having the need of dose adaption of your anticoagulation medication in atrial fibrillation?

1. Positive
2. Negative
8. Not sure
9. Decline to answer

BASE: ALL RESPONDENTS WITH Q8_2.4=1

Q8_2.4_1 Why do you think that it will be positive for you?

____

BASE: ALL RESPONDENTS WITH Q8_2.4=2

Q8_2.4_2 Why do you think that it will be negative for you?

____

BASE: ALL RESPONDENTS TAKING PRESCRITION MEDICATIONS (Q8_1/1-96)

Q8_3 In the past 2 years, how often have any of your doctors or your pharmacists reviewed and discussed all the different medications you are using, including medicines prescribed by other doctors?

1. Always
2. Often
3. Sometimes
4. Rarely or never
8. Not sure
9. Decline to answer

BASE: ALL RESPONDENTS TAKING PRESCRIPTION MEDICATIONS (Q8_1/1-97)

Q8_4 In the past 2 years, were you ever told by a pharmacist that the prescription you were about to fill might be harmful because of other medications you were taking?

1. Yes
2. No
8. Not sure
9. Decline to answer

BASE: ALL RESPONDENTS TAKING PRESCRIPTION MEDICATIONS (Q8_1/1-97)

Q8_5 In the past two years, have you ever skipped or stopped taking prescription medicine for any reason before you were supposed to, including for cost?

1. Yes
2. No
8. Not sure
9. Decline to answer

BASE: ALL RESPONDENTS WHO STOPPED TAKING MEDICATION (Q8_5/1)

Q8_6 Did you tell your doctor or nurse that you did not take, or stopped taking the medication?

1. Yes
2. No
8. Not sure
9. Decline to answer

SECTION 9: MEDICAL ERRORS/SAFETY ISSUES

BASE: ALL RESPONDENTS

Q9_1 Have you ever been given the wrong medication or wrong dose by a doctor, nurse, hospital or pharmacist when filling a prescription at a pharmacy or while hospitalized in the past 2 years?

1. Yes
2. No
8. Not sure
9. Decline to answer

BASE: ALL RESPONDENTS

Q9_2 Have you believed a medical mistake was made in your treatment or care in the past 2 years?

1. Yes
2. No
8. Not sure
9. Decline to answer

BASE: ALL RESPONDENTS

Q9_3 Have you had any blood tests, x-rays, or any other medical tests in the past 2 years?

1. Yes
2. No
8. Not sure
9. Decline to answer

BASE:ALL RESPONDENTS WHO HAVE HAD TEST ORDERED IN PAST 2 YEARS (Q9_3/1)

Q9_4 In the past 2 years, have you

A Been given incorrect results for a diagnostic or lab test, this could include getting someone else's test results
B Experienced delays in being notified about abnormal test results

1. Yes
2. No
8. Not sure
9. Decline to answer

BASE: ALL RESPONDENTS WITH WRONG MEDICATION/DOSE (Q9_1/1) OR MEDICAL MISTAKE (Q9_2/1) OR LAB MISTAKE (9_4/1-2 ANY)

Q9_5 Did the most recent mistake, medication error, or diagnostic test error occurs while you were hospitalized?

1. Yes
2. No
8. Not sure
9. Decline to answer

BASE: ALL RESPONDENTS WITH WRONG MEDICATION/DOSE (Q9_1/1) OR MEDICAL MISTAKE (Q9_2/1) OR LAB MISTAKE (9_4/1-2 ANY)

Q9_6 Did this mistake, medication error, or diagnostic test error cause a very serious health problem, somewhat serious health problem, not serious health problem, or no health problem at all?

1. Very serious problem
2. Somewhat serious problem
3. Not serious problem
4. No problem at all
8. Not sure
9. Decline to answer

SECTION 10: EXPERIENCES WITH CARE IN HOSPITAL

BASE: ALL RESPONDENTS WHO WERE HOSPITALIZED (Q2_7/1)

Q10_1 You mentioned that you were hospitalized in the past 2 years. For the next few questions, I want you to think only about the care you received while you were admitted as an inpatient that is, stayed as a patient overnight.

BASE: ALL RESPONDENTS WHO WERE HOSPITALIZED (Q2_7/1)

Q10_2 While in the hospital, did you develop an infection?

1. Yes
2. No
8. Not sure
9. Decline to answer

BASE: ALL RESPONDENTS WHO WERE HOSPITALIZED (Q2_7/1)

Q10_3 When you left the hospital

A. Did you receive clear instructions about symptoms to watch for and when to seek further care?
B. Did you know who to contact if you had a question about your condition or treatment
C. Did the hospital staff provide you with a written plan for your care after discharge?
D. Did the hospital make arrangements for you to have follow-up with a doctor or other health care professional

1. Yes
2. No
8. Not sure
9. Decline to answer

BASE: ALL RESPONDENTS WHO WERE HOSPITALIZED (Q2_7/1)

Q10_4 When you left the hospital, were you given any new prescription medications?

1. Yes
2. No
8. Not sure
9. Decline to answer

BASE: ALL RESPONDENTS WHO WERE GIVEN NEW RX IN HOSPITAL (Q10_4/1)

Q10_5 Did someone discuss with you what to do about other medications you were using before you were hospitalized?

1. Yes
2. No
3. Not taking any medications before hospitalized
8. Not sure
9. Decline to answer

BASE: ALL RESPONDENTS WHO WERE HOSPITALIZED (Q2_7/1)

Q10_6 After you were discharged, were you readmitted to a hospital or did you have to go to a hospital emergency department as a result of complications that occurred during your recovery?

1. Yes, readmitted to a hospital
2. Yes, had to go to the ER
3. Yes, both
4. No / Had no complications
8. Not sure
9. Decline to answer

SECTION 11: EMERGENCY ROOM USE

BASE: ALL RESPONDENTS

Q11_1 How many times have you personally used a hospital emergency department in the past 2 years?

[Range 00-96, 98=not sure, 99=decline to answer]

BASE: ALL RESPONDENTS WHO HAVE USED ER (Q11_1/1-97)

Q11_2 The last time you went to the hospital emergency department, was it for a condition that you thought could have been treated by your regular doctor if he or she had been available?

1. Yes
2. No
8. Not sure
9. Decline to answer

SECTION 12: CHRONIC ILLNESS CARE

BASE: ALL RESPONDENTS

Q12_1 Have you ever been told by a doctor that you have

( ) A Hypertension, sometimes called high blood pressure
( ) B Heart disease, including heart attack
( ) C Diabetes
( ) D Arthritis
( ) E Asthma, COPD, or any other chronic lung problems
( ) F Depression, anxiety or other mental health problems
( ) G Cancer

1. Yes
2. No
8. Not sure
9. Decline to answer

BASE: ALL RESPONDENTS

Q12_2 Which of following risk factors apply to you?

A. Congestive/Chronic heart failure/Left ventricular dysfunction
B. Hypertension
C. Age over 75
D. Diabetes mellitus
E1. Stroke
E2. TIA [transient ischemic attack]
E3. Thrombo-embolism
F. Vascular disease
G. Age 65-74
H. Sex category
J. Abnormal renal function
K. Abnormal liver function
L. Stroke
M. Bleeding
N. Labile INRs
O. Elderly (over 65)
P. Use of “illegal” Drugs
Q. Alcohol

1. Yes
2. No / Not applicable
8. Not sure
9. Decline to answer

BASE: ALL RESPONDENTS

Q12_3_1 What kind of atrial fibrillation are you suffering from?

1. Paroxysmal
2. Persistent
3. Long-standing persistent
4. Permanent
8. Not sure
9. Decline to answer

BASE: ALL RESPONDENTS

Q12_3_2 Is this the first time you have been diagnosed with atrial fibrillation?

1. Yes
2. No
8. Not sure
9. Decline to answer

BASE: ALL RESPONDENTS

Q12_3_3 When was atrial fibrillation diagnosed for the first time?

1. ≤ one year ago (one or less than a year)
2. ≤ 5 years ago(2 -5 years ago)
3. ≤ 10 years ago (6-10 years ago)
4. >10 years ago (More than 10 years ago)
8. Not sure
9. Decline to answer

BASE: ALL RESPONDENTS

Q12_4_1 Do you have an anticoagulation or treatment card?

1. Yes
2. No
8. Not sure
9. Decline to answer

BASE: ALL RESPONDENTS WHO ARE Q12_4_1 / 1

Q12_4_2 Is your PT/INR (Prothrombin Time/International Normalized Ratio) value tested regularly?

1. Yes Number of times: ____________________________
2. No
8. Not sure
9. Decline to answer

BASE: ALL RESPONDENTS WHO ARE Q12_4_2 / 1

Q12_4_3 What was your most recent INR value?

1.	An INR value less than 2.0	(Value INR < 2.0)
2	INR value between 2.0-2.5	(Value INR 2.0–2.5)
3.	INR value between 2.6 - 3.0	(Value INR of 2.6–3.0)
4.	INR value between 3.1-3.5	(Value INR 3.1–3.5)
5.	INR value higher than 3.5	(Value INR >3.5)
8.	Not sure	
9.	Decline to answer	

BASE: ALL RESPONDENTS WHO ARE Q12_4_2 / 1

Q12_4_4 Where is your PT/INR monitored?

1. At the hospital
2. At the general practitioner’s practice
3. At the specialist’s practice
4. At the community pharmacy
5. At home, performed by a district nurse
6. At home, performed by me
7. Other, please specify
8. Not sure
9. Decline to answer

BASE: ALL RESPONDENTS WHO ARE Q12_4_2 / 1

Q12_4_5 What do you think of the possibility of no longer having the need to get coagulation tests done like PT/INR?

1. Positive
2. Negative
8. Not sure
9. Decline to answer

BASE: ALL RESPONDENTS WHO ARE Q12_4_5 / 1

Q12_4_5_1 Why would it be positive for you?

1. Saves time
2. Saves money
3. No need to go to anywhere for the test
4. No coordination problems any more
5. Other, please specify
8. Not sure
9. Decline to answer

BASE: ALL RESPONDENTS WHO ARE Q12_4_5 / 2

Q12_4_5_2 Why would it be negative for you?

1. I need to know my INR/PT value
2. Don't see my doctor on a regular base any more
3. Uncertainty
4. Other, please specify
8. Not sure
9. Decline to answer

BASE: ALL RESPONDENTS

Q12_5 Have you undergone any of the following procedures?

A. Cardioversion with drugs; number of times: ____________________________
B. Electrical cardioversion; number of times: ____________________________
C. Catheter ablation; number of times: ____________________________
D. Pacemaker
E. Surgical ablation
F. Other cardiac device implanted

1. Yes
2. No
8. Not sure
9. Decline to answer

BASE: ALL RESPONDENTS

Q12_6 What in addition could the healthcare system do to support you in the management of your disease atrial fibrillation?

1 Information e.g. brochures
2. Information provided by physician
3. Information provided the internet
4. Communication with physician
5. Exchange opportunities with other patients
6. Support for private life (e.g. help in household etc.]
7. Financial support
10. Other, please specify
8. Not sure
9. Decline to answer

SECTION 13: RATING OF OVERALL CARE

BASE: ALL RESPONDENTS

Q13_1 Overall, how do you rate the quality of medical care that you have received in the past 12 months?

1. Excellent
2. Very good
3. Good
4. Fair
5. Poor
8. Not sure
9. Decline to answer

BASE: ALL RESPONDENTS (Q2_9 1, 3, 4, 5, 6)

Q13_2 Now I would like to ask you a few questions about your experience using the Internet or email for your health care needs. Do you have access to the Internet?

1. Yes
2. No
8. Not sure
9. Decline to answer

BASE: ALL RESPONDENTS (Q2_9/2)

Q13_3 Does any another person in your household, have access to the Internet?

1. Yes, I personally have access to the Internet
2. No, I do not have access to the Internet, but another person in my household does
3. No, no one in my household has access to the Internet
8. Not sure
9. Decline to answer

BASE: SP, FR, IT, UK RESPONDENTS WHO HAVE ACCESS TO INTERNET (Q2_9 1, 3, 4, 5, 6 and Q13_2/1)

Q13_4 Have you ever used the Internet to look for health or illness information for yourself, a family member or friend?

1. Yes
2. No
8. Not sure
9. Decline to answer

BASE: GERMANY QUALIFIED RESPONDENTS WHO HAVE ACCESS TO INTERNET (Q2_9/2 & Q13_3/1)

Q13_5 Have you ever used the Internet to look for health or illness information for yourself, a family member or friend?

1. Yes, have looked for information for myself
2. Yes, have looked for information for someone else
3. Yes, have looked for information for both myself and someone else
4. No, have not looked for health information on the Internet
8. Not sure
9. Decline to answer

BASE: ALL RESPONDENTS (Q2_9/2)

Q13_6 Have any of your family members or friends ever given you information from the Internet about your illness or health problems?

1. Yes
2. No
8. Not sure
9. Decline to answer

BASE: ALL RESPONDENTS WITH ACCESS TO INTERNET (Q13_2/1 or Q13_3/1) AND REG DOC/GP/PLACE OF CARE (Q5_1/1 or Q5_2/1)

Q13_7 Can you communicate with your doctor by email?

1. Yes
2. No
8. Not sure
9. Decline to answer

BASE: ALL RESPONDENTS WITH ACCESS TO INTERNET (Q13_2/1 or Q13_3/1)

Q13_8 Can you currently do any the following

Make a doctor's appointment via the Internet?
Refill a prescription online?
Access your own medical record online?

1. Yes
2. No
8. Not Sure
9. Decline to answer

SECTION 14: HEALTH CARE COVERAGE

BASE: ALL RESPONDENTS

Q14_1 In addition to government funded health services, are you currently covered by any private health insurance that you or your family pays for or that an employer or association provides?

1. Yes, have private insurance
2. No, do not have private insurance
8. Not sure
9. Decline to answer

BASE: ALL RESPONDENTS

Q14_1_1 Are you under a plan where the whole of your health costs are covered by the national insurance fund because you have a chronic illness?

1. Yes
2. No
8. Not sure
9. Decline to answer

BASE: ALL RESPONDENTS

Q14_2 What kind of health insurance do you have?

1. National health insurance without any private insurance
2. National insurance and also private insurance
3. Private insurance only
4. Insured through welfare
5. No health insurance
8. Not sure
9. Decline to answer

BASE: ALL RESPONDENTS

Q14_3 In the past year, was there ever a time when you did not have any health insurance?

1. Yes
2. No
8. Not sure
9. Decline to answer

BASE: ALL RESPONDENTS

Q14_4 In the past 3 years, how many times you have changed health insurance or health plans?

1. Not at all
2. One time
3 2 or more times
8. Not sure
9. Decline to answer

BASE: ALL RESPONDENTS

Q14_5 In the past 12 months, has a doctor told you he or she would not prescribe a medication for you because your insurance would not pay for it or because of non-reimbursement?

1. Yes
2. No
8. Not sure
9. Decline to answer

BASE: ALL RESPONDENTS

Q14_6 In the past 2 years, was there ever a time when you had to contact your insurance company because they did not pay a bill promptly or denied payment?

1. Yes
2. No
3. Not applicable
8. Not sure
9. Decline to answer

SECTION 15: OUT OF POCKET COSTS

BASE: ALL RESPONDENTS

Q15_1 In the past 12 months, about how much have you and your family spent out-of-pocket for medical treatments or services that were <u>not</u> covered by insurance?

This would include the charges for prescription medicines, and for medical care treatments or tests by a doctor or another health professional. This includes also expenditures due to illness e.g. for nursing service, taxi to medical care or physician, changes for home / household (e.g. lift), house keeper etc.
(Range: 0-999997, 999998 = not sure, 999999=decline to answer)

BASE: ALL RESPONDENTS WHO HAVE IN Q15_1 RANGE >0 AND NOT 999998 OR 999999

Q15_2_ How much of these out-of-pocket costs are specifically for your Atrial Fibrillation treatment?
(Range: 0-999997, 999998 = not sure, 999999=decline to answer)

BASE: ALL RESPONDENTS WHO HAVE IN Q15_2 RANGE >0 AND NOT 999998 OR 999999

How much was for:

Q15_2_1 Co-payment for Atrial Fibrillation prescription medicine?
(Range: 0-999997, 999998 = not sure, 999999=decline to answer)

Q15_2_2_ Medical care for Atrial Fibrillation e.g. co-payment
(Range: 0-999997, 999998 = not sure, 999999=decline to answer)

Q15_2_3 Test by a doctor or other healthcare professional due to your Atrial Fibrillation
(Range: 0-999997, 999998 = not sure, 999999=decline to answer)

Q15_2_4_ Other medical treatments or services that were not covered by insurance but necessary due to your Atrial Fibrillation? Such as expenditures due to illness e.g for nursing service, taxi to medical care or physician, changes for home / household (e.g. lift), house keeper etc.
(Range: 0-999997, 999998 = not sure, 999999=decline to answer)

SECTION 16: DEMOGRAPHICS

BASE: ALL RESPONDENTS

Q16_1 Were you born in [Q2_10 country] or somewhere else?

1. Yes, born in this country
2. No, not born in this country
8. Not sure
9. Decline to answer

BASE: ALL RESPONDENTS

Q16_2 Were your parents born in [Q2_9 country] or somewhere else?

1. Yes, both parents were born in [Q2_9 country]
2. No, both parents were born in some other country
3. One parent was born in [Q2_9 country] and the other was born in some other country
8. Not sure
9. Decline to answer

BASE: ALL RESPONDENTS

Q16_3 Including you, how many adults, 18 or older, live in your household?
[Range 01-20, 98=not sure, 99=decline to answer]

BASE: ALL RESPONDENTS

Q16_4 Which of these best describes you? Are you married, not married but living with a partner, divorced, separated, widowed, or single and never married?

01. Married
02. Living with a partner
03. Divorced
04. Separated
05. Widowed
06. Single, never married
98. Not sure
99. Decline to answer

BASE ALL RESPONDENTS

Q16_5 What is the highest level of education you have completed to date?

01. Primary School
02. Secondary School with Brevet Diploma
03. Secondary, technical or vocational school with Baccalaureate or any equivalent
04. High school graduate or equivalent (e.g., GED)
05. Completed some college, but no degree
06. Completed technical or community college (e.g., associate’s degree)
07. College or university degree or higher
08 No graduation
98. Not sure
99. Decline to answer

BASE: ALL RESPONDENTS

Q16_6 The average household income of families in this country is around [Enter amount] a year. By comparison, is your household income…?

1. Much above average
2. Somewhat above average
3. Average
4. Somewhat below average
5. Much below average
8. Not sure
9. Decline to answer

BASE: ALL RESPONDENTS

Q16_7 Which of the following describes where you live?

1. City/large town
2. Suburbs of a city/large town
3. Small town
4. Village or rural location
8. Not sure
9. Decline to answer

BASE: ALL RESPONDENTS

Q16_8 Region for each country

BASE: ALL RESPONDENTS

That completes all the questions we have for you today. Thank you very much for your participation in this important survey. We truly appreciate your feedback. Thank you for your time today.
